# Long-Term Survival of Patients With Chemotherapy-Naïve Metastatic Nasopharyngeal Carcinoma Receiving Cetuximab Plus Docetaxel and Cisplatin Regimen

**DOI:** 10.3389/fonc.2020.01011

**Published:** 2020-06-19

**Authors:** Mengping Zhang, He Huang, Xueying Li, Ying Huang, Chunyan Chen, Xiaojie Fang, Zhao Wang, Chengcheng Guo, Sioteng Lam, Xiaohong Fu, Huangming Hong, Ying Tian, Taixiang Lu, Tongyu Lin

**Affiliations:** ^1^State Key Laboratory of Oncology in South China, Collaborative Innovation Center for Cancer Medicine, Department of Medical Oncology, Sun Yat-sen University Cancer Center, Guangzhou, China; ^2^Department of Oncology, The First Affiliated Hospital of Sun Yat-sen University, Guangzhou, China; ^3^Department of Medical Oncology, The Seventh Affiliated Hospital, Sun Yat-sen University, Shenzhen, China; ^4^State Key Laboratory of Oncology in South China, Collaborative Innovation Center for Cancer Medicine, Department of Radiation Oncology, Sun Yat-sen University Cancer Center, Guangzhou, China; ^5^Centro Hospitalar Conde de Sáo Januário, Macau, China; ^6^Department of Oncology, Shenzhen Nanshan Hospital, Shenzhen, China

**Keywords:** survival, chemotherapy, metastatic nasopharyngeal carcinoma, cetuximab, induction therapy

## Abstract

**Purpose:** Metastatic nasopharyngeal carcinoma (mNPC) remains incurable. This prospective study aimed to investigate whether adding cetuximab to cisplatin-based induction therapy could improve efficacy and survival for chemotherapy-naïve mNPC patients.

**Patients and Methods:** Eligible chemotherapy-naïve mNPC patients were enrolled, including those initially diagnosed with mNPC (IM) and those with first-relapse metastases after radiotherapy (RM). Patients all received induction chemotherapy (IC) including docetaxel and cisplatin plus cetuximab. Those who obtained objective remission after IC would continue to receive radiotherapy concurrent with cetuximab and cisplatin, and further capecitabine as maintenance. Contemporaneous patients who received conventional therapy served as controls.

**Results:** Forty-three patients were enrolled, including 17 IM and 26 RM patients. Thirty-nine (90.7%) patients had WHO III subtype. The overall response and complete response (CR) rates were, respectively, 79.1 and 34.9% after induction therapy and 76.7 and 46.5% after chemoradiotherapy. The 5-year overall survival (OS) and progression-free survival (PFS) rates reached 34.9 and 30%, respectively. Subgroup analysis showed that compared with RM patients, IM patients had a higher 5-year OS (58.8 vs. 19.2%) and PFS (52.9 vs. 19.2%). The IM group had a higher CR rate of induction treatment than the RM group (52.9 vs. 23.1%). No treatment-related death was observed. Twelve patients (27.9%) remained alive with disease-free survival times from 60+ to 135+ months. Control patients showed a substantially lower survival rate (5-year OS, 10.9%) and few long-term survivors.

**Conclusions:** This regimen resulted in significantly improved efficacy and survival, which indicates a potentially curative role for chemotherapy-naïve mNPC, especially in newly diagnosed patients. A phase III clinical trial (NCT02633176) is ongoing for confirmation.

## Introduction

Nasopharyngeal carcinoma (NPC) is epidemic in southern China and Southeast Asia (1). Additionally, ~25–30% of NPC patients exhibit metastatic disease (2), and 15% of all NPC patients present with distant metastases at primary diagnosis (3). The outcomes of patients with metastatic NPC (mNPC) are heterogeneous, and long-term survival is possible in very few patients (4). On the basis of high-level evidence, patients with recurrent or primary mNPC generally have very poor survival, with a median overall survival of 11.5–15 months reported 10 years ago (5, 6) and a median survival of 29.1 months reported in 2016 (7). Generally, mNPC is recognized as an incurable disease, as few patients survive beyond 5 years.

Platinum-containing doublet regimens or concurrent chemoradiotherapy (CCRT) alone or induction chemotherapy followed by chemoradiotherapy continue to be regarded as standard first-line treatments for patients with recurrent or metastatic NPC. Gemcitabine, capecitabine, paclitaxel, and docetaxel have also been combined with cisplatin and yield similar survival (8, 9). However, no randomized trials have defined the optimum regimens.

Cetuximab is an IgG1 monoclonal antibody that inhibits ligand binding to the epidermal growth factor receptor (EGFR) (10). EGFR expression is reported in more than 85% of undifferentiated NPCs and is associated with a poor clinical outcome (11). Radiotherapy and platinum-based chemotherapy plus cetuximab have enhanced activity against head and neck cancer, with improved overall survival (OS) (12, 13). Although distinct differences exist between NPC and other head and neck cancers, despite originating from a similar cell or tissue lineage, we speculated that adding an EGFR inhibitor to platinum-based chemotherapy and CCRT could be beneficial for mNPC. Moreover, a phase 2 study of cetuximab in combination with a cytotoxic agent showed clinical activity and an acceptable safety profile in heavily pretreated patients with mNPC (14).

A meta-analysis of 11 randomized trials showed that longer first-line chemotherapy is associated with longer OS (15). However, prolongation of docetaxel or cisplatin exposure until disease progression is unrealistic because of cumulative toxic effects. Therefore, switching to a more tolerable chemotherapy, such as capecitabine, as a maintenance regimen might be a more effective treatment strategy.

We therefore conducted this single-center, prospective study of an epidermal growth factor receptor antibody (cetuximab)-containing induction therapy and chemoradiotherapy regimen to investigate whether it would significantly improve survival outcomes while maintaining tolerability in mNPC patients without prior systemic therapy and would alter the therapeutic modality from conventional palliative to curative treatment.

## Methods

### Study Design and Patients

We performed an investigator-initiated, open-label, single arm, single center, phase 2 trial at Sun Yat-sen University Cancer Center, Guangzhou, China. Eligible participants were 18 to 65 years of age and had histologically confirmed mNPC, including initial diagnosed NPC with metastases (IM) and first-relapse metastases after curative radiotherapy without neoadjuvant or adjuvant chemotherapy (RM). Pretreatment staging and metastases were confirmed via positron emission tomography/computerized tomography scans (PET/CT). Eligible patients had a type II or III histological subtype according to the WHO classification. Other eligibility criteria were as follows: patients had not received any previous systemic chemotherapy for recurrent or metastatic disease; had an Eastern Cooperative Oncology Group (ECOG) performance status of 0 or 1; had not received previous treatment with any investigational drug, surgery, irradiation or other anticancer therapies within the prior 4 weeks; had no known brain metastases; had adequate organ function as defined by adequate bone marrow function (hemoglobin≥90 g/L, WBC count≥3 × 10^9^/L, platelet count≥100 × 10^9^/L), renal function (serum creatinine ≤ 140 μmol/L or calculated creatinine clearance≥40 mL/min), and liver function (ALT or AST ≤ 3 × the upper limit of normal, bilirubin ≤ 2 × the upper limit of normal); had no uncontrolled cardiac or other disease with life expectancy of 3 months or more; provided written informed consent; and was amenable for regular follow-up. The study protocol was approved by the ethics committee of Sun Yat-sen University Cancer Center.

### Procedures

The induction chemotherapy regimen was repeated every 3 weeks and comprised the following: intravenous docetaxel 75 mg/m^2^ day 1; cisplatin at 25 mg/m^2^ on days 1, 2, and 3; and cetuximab at 250 mg/m^2^ on days 0, 7, and 14 with an initial dose of 400 mg/m^2^. This induction regimen was followed by CCRT consisting of intensity-modulated radiotherapy (IMRT) plus concomitant cetuximab (250 mg/m^2^/week for 6 cycles) and cisplatin (75 mg/m^2^/3 weeks for 2 cycles). IMRT was given at 68–70 Gy over 30 daily fractions over 6 weeks to the planning target volume of the existing primary tumor in IM patients, or 64–66 Gy in RM patients with previous radiotherapy, with additional radiotherapy of 62–66 Gy over 30 fractions to metastatic regional neck nodes if indicated. After CCRT, capecitabine was continued as maintenance therapy (cycles were repeated every 21 days with 1,000 mg/m^2^ twice daily, days 1 through 14).

Patients received this induction therapy regimen for a maximum of six cycles or until disease progression, death, intolerable toxicities, or patient request to stop. Furthermore, only patients who obtained complete or partial responses (CR or PR) after induction therapy could receive CCRT. For patients with locoregional metastatic bone lesions, additional radiotherapy with 30–40 Gy in 10–20 fractions to these sites of lesions was performed. Patients with other residual metastatic foci in lung, liver, and non-cervical lymph nodes after induction therapy that was amenable to local therapy were offered surgery or radiofrequency ablation before CCRT. For patients who exhibited a CR after CCRT, maintenance therapy was continued for up to 3 years or until unacceptable toxicity, disease progression, or death.

Treatment-emergent adverse events (AEs) were assessed with the Common Terminology Criteria for Adverse Events version 3.0 and were noted separately for the induction, CCRT, and maintenance treatment. The indications for cetuximab dose adjustment or interruption were described previously (14). The chemotherapy was continued independent of any temporary interruption of cetuximab. Cetuximab was not withheld for chemotherapy-related toxicities, unless the patient developed a concomitant illness that, in the opinion of the investigator, mandated interruption of therapy.

Tumor response was assessed by CT imaging according to RECIST version 1.1 by the independent image committee every two cycles during induction therapy and every 3 weeks during CCRT. CR and PR were defined, respectively, as 100% or at least 30% decrease in the sum of the longest diameters of target lesions compared with baseline. Follow-up was performed at the outpatient clinic every 1–3 months for the first year, every 3 months for the second year, every 6 months for the third to fifth years, and annually thereafter.

### Outcomes

The primary objective was to determine progression-free survival (PFS), which was defined as the time from treatment initiation to disease progression or death from any cause, whichever came first. Secondary endpoints included the proportion of patients who had a confirmed overall response (OR) (defined as CR or PR lasting at least 4 weeks according to the RECIST 1.1), OS (defined as the time from treatment initiation to the date of death or last follow-up), and AEs. Patients were considered long-term survivors if they were disease-free for a period of more than 60 months without any treatment except maintenance treatment after a CR.

### Statistical Analyses

The asymptotic distribution, provided in Lachin [(16), p. 409–411] was used to calculate the sample size for this single arm trial. The justification for the sample size is explained below. The two-sides Type I error rate was set at 5%, and the type II error rate set at 20%, giving 80% power. The accrual period was set at 1 year, and the total study period was set at 2 years. The OS rate at 1 year, based upon a previous study (17), is as high as 60% among patients treated with platinum-based therapy. Among patients receiving the novel regimen, the 1-year OS rate was expected to increase to 80%. This difference of 20% equates to a hazard ratio of 0.44. The sample size calculation, given the above information, estimates that 12 events were needed. Finally, it was estimated that 25 patients were required to achieve this number of events allowing for a 10% loss to follow-up/non-adherence rate.

PFS and OS were estimated using the Kaplan-Meier method. Hazard ratios were calculated by the use of the Cox proportional-hazards model. The response rate and its 95% CI (using the method of Pearson and Clopper) were calculated. We performed subgroup analyses among subgroups between mNPC patients with IM and RM for OS and PFS and response rate. We performed *post-hoc* subgroup analyses for OS and PFS, focusing on CR after induction therapy. We calculated the median follow-up time as the median of all enrolled patients, irrespective of whether the patients had died (18). Descriptive statistics were used for safety evaluations. All statistical testing was two-sided at the nominal 5% significance level. All analyses were performed with SPSS 13.0.

## Results

Between July 2006 and December 2014, we enrolled 43 patients, 17 (39.5%) with initial diagnosis of NPC with metastases (IM) and 26 (60.5%) with first-relapse metastases (RM). All patients had evidence of EGFR-positive NPC. Table 1 summarizes the baseline characteristics of all 43 enrolled patients.

**Table 1:** Demographic and clinical characteristics.

**Characteristics**	**NO. (%)**
NO.	43
Gender	
Female	7 (16.3)
Male	36 (83.7)
Age, years^*^	
Median	43
Range	23-63
ECOG performance status	
0	14 (32.6)
1	29 (67.4)
Histology	
WHO type 2	4 (9.3)
WHO type 3	39 (90.7)
EBV-DNA status	
Positive^*^	32 (74.4)
Negative	11 (25.6)
Number of metastatic organs	
1	27 (62.8)
2	8 (18.6)
≥3	8 (18.6)
Sites of disease at registration	
Distant lymph node	7 (16.3)
Bone	32 (74.4)
Liver	14 (32.6)
Lung	11 (25.6)
Others	6 (14.0)
Prior radiotherapy	
Yes	26 (60.5)
No	17 (39.5)

*Data are presented as a number (percentage) unless otherwise indicated*.

* *Positive: EBV- DNA copies ≥10^3^ copies/mL*.

After the completion of induction chemotherapy, median cycles given to patients were 5 cycles (IQR 4–6). The OR rate was 79.1%, and 15 of 43 patients (34.9%) had a CR at all disease sites. Cetuximab was interrupted in 5 patients (11.6%) due to grade 3 acneiform skin rash. Six (13.9%) required a dose reduction of cisplatin or docetaxel during induction therapy due to serious myelosuppressive toxicity. Thirty-four patients obtained a CR or PR after induction chemotherapy, including 16 IM patients and 18 RM patients, and went on to receive CCRT; the OR and CR rates after CCRT were 76.7 and 46.5%, respectively (Table 2). Due to drug-related toxicity or patient refusal, only 15 patients received capecitabine as maintenance following CR after CCRT, among which 5 patients had disease progression during this period.

**Table 2:** Antitumor efficacy.

**Variable**	**IM (*n* = 17)**	**RM (*n* = 26)**	**Overall (*n* = 43)**
**Response after induction chemotherapy**, ***n*** **(%)**			
Complete response	9 (52.9)	6 (23.1)	15 (34.9)
Partial response	7 (41.2)	12 (46.2)	19 (44.2)
Stable disease	0 (0)	6 (23.1)	6 (14.0)
Progressive disease	1 (5.9)	2 (7.7)	3 (7.0)
Overall response, % [95% CI])	94.1 [82.9–100]	69.2 [51.5–87]	79.1 [66.9–91.2]
Disease control	94.1 [82.9–100]	92.3 [82.1–100]	93 [85.4–100]
**Response after chemoradiotherapy**, ***n*** **(%)**			
Complete response	12 (70.6)	8 (30.8)	20 (46.5)
Partial response	4 (23.5)	9 (34.6)	13 (30.2)
Stable disease	0 (0)	6 (23.1)	6 (14.0)
Progressive disease	1 (5.9)	3 (11.5)	4 (9.3)
**Overall survival**			
Median, months [95% CI]	Unreached^*^	20.3 [13.3–37.6]	32.9 [18.2–47.5]
2-year rate, %	88.2	42.3	60.5
5-year rate, %	58.8	19.2	34.9
**Progression-free survival**			
Median, months [95% CI]	Unreached^*^	12.5 [7.9–17.1]	18.3 [10.6–26.0]
2-year rate, %	58.8	30.8	41.9
5-year rate, %	52.9	19.2	30.0

* *Indicates that the IM subgroup significantly differed from RM subgroup; CI, confidence interval*.

**Figure 1:**
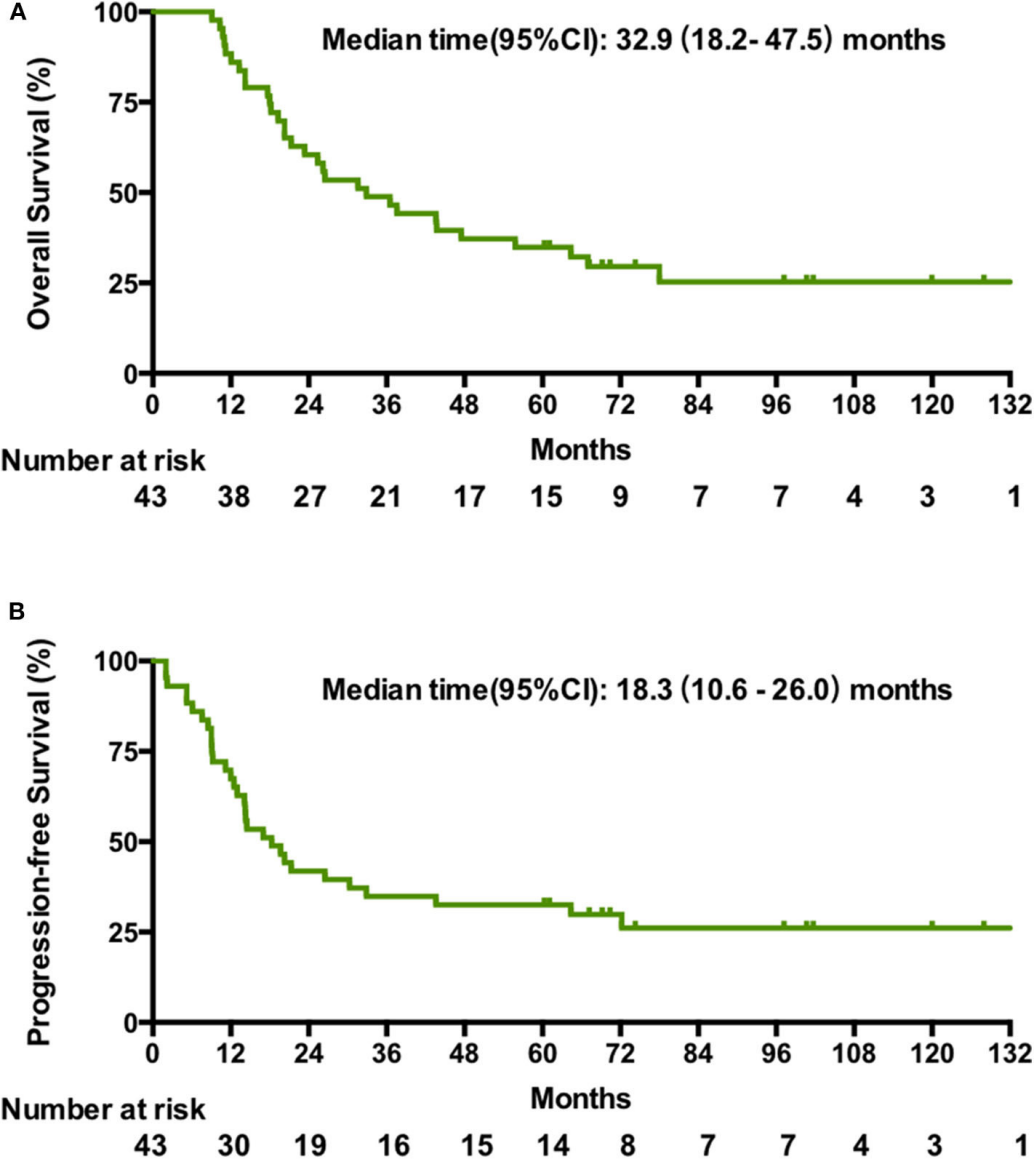
Kaplan-Meier estimates of the overall OS **(A)** and PFS **(B)** in patients treated with the novel regimen.

The cutoff date for survival analysis was July 30, 2018. The median follow-up time for survival was 89 months (range, 32–135). During follow-up, 31 patients had disease progression and finally died. After documented SD or PD during treatment or follow-up period, patients received second-line or third-line chemotherapy or palliative radiotherapy or did not receive any antitumor therapy. The median OS was 32.9 months (95% CI, 18.2–47.5). Kaplan-Meier estimated OS rates at 6 months, 1, 2, 3, and 5 years were 100, 86, 60.5, 46.4, and 34.9% respectively (Figure 1A). The median PFS was 18.3 months (95% CI, 10.6–26 months). The PFS at 6 months, 1, 2, 3, and 5 years was 86, 67.4, 41.9, 34.9, and 30% respectively (Figure 1B). Contemporaneous patients in the same hospital received conventional regimen showed poorer survival: for OS, median OS, 21 mo, 95% CI, 17.8–24.0, HR = 2.1, 95% CI, 1.3–3.3; for PFS, median PFS, 8 mo, 95% CI, 6.4–9.6 mo, HR = 3.3, 95% CI, 2.1–5.3 (Supplement Figure 1). The baseline data of the two groups were comparable which were showed in the Supplement Table 1.

**Figure 2:**
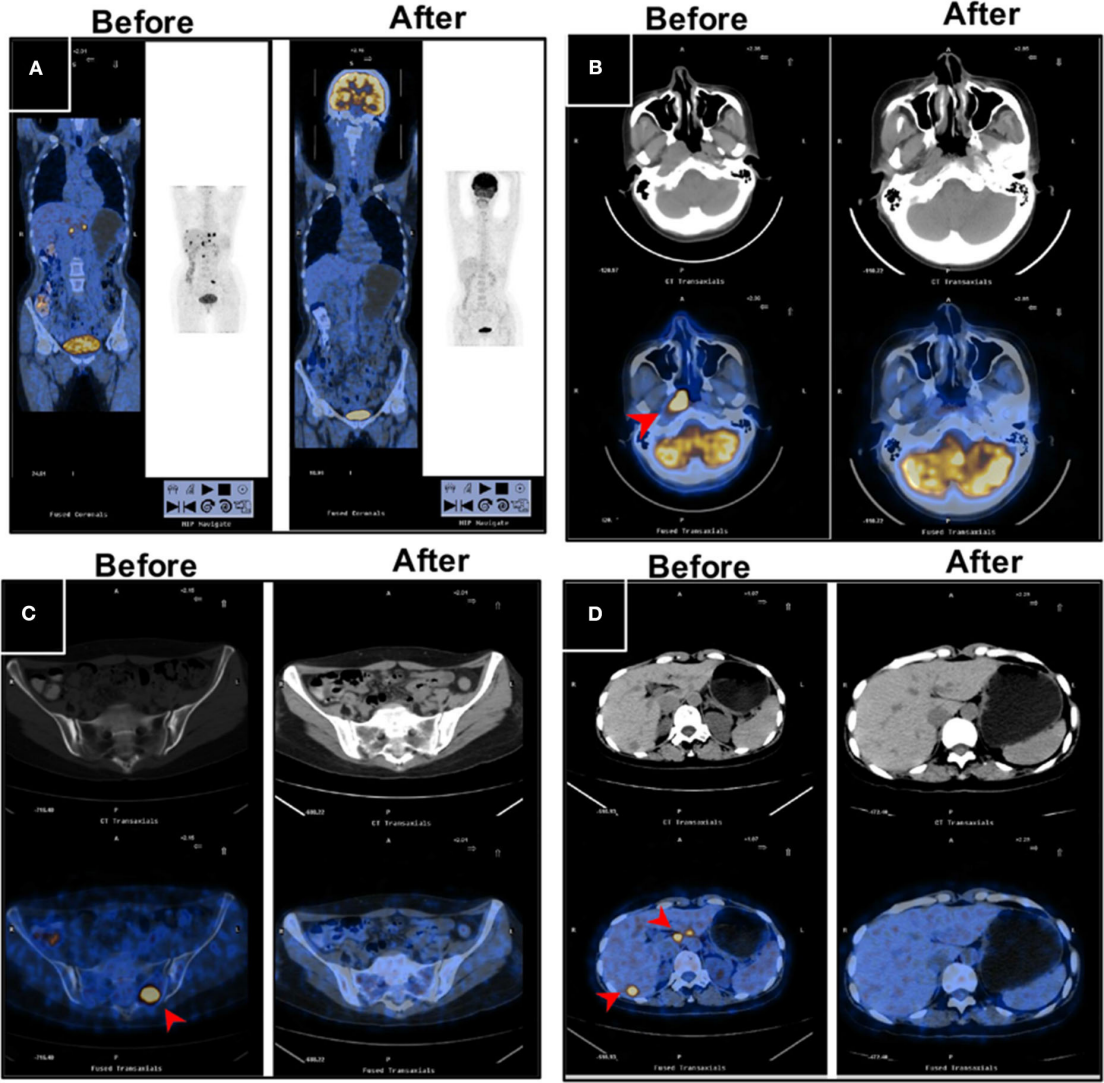
PET/CT images for a long-term disease-free patient before and after the novel regimen. The female patient, 48 years of age, with an initial diagnosis of nasopharyngeal carcinoma with bone and liver metastases, EBV+, survived without disease for more than 120 months. **(A)** The systemic lesions, **(B)** the primary nasopharyngeal tumors, **(C)** the bone metastases, and **(D)** the liver metastases disappeared or decreased after treatment compared with before treatment.

With regard to the cutoff date, there were 15 long-term survivors who were disease-free for more than 60 months without treatment after obtaining a CR during the novel treatment. Among these 15 patients, 12 patients were still alive with no evidence of disease after treatment with a disease-free survival time from 60+ to 135+ months, as shown in Table 3 and Figure 2; two patients died of disease progression while in CR at 64 and 72 months after treatment; and one patient died of acute leukemia at 64 months after treatment.

**Table 3:** Characters and survival outcome of long-term disease-free survivors.

**Patient**	**Gender**	**Age**	**Group**	**Metastatic sites**	**EBV status**	**Response of introduction treatment**	**Disease-free survival time (months)**
1	Male	63	IM	Bone	Negative	CR	102
2	Female	48	IM	Bone, liver, lung	Positive	CR	120
3	Female	43	IM	Lung	Negative	CR	61
4	Female	63	IM	Bone, distant lymph node, pelvic	Positive	CR	70
5	Female	46	IM	Bone	Negative	CR	69
6	Male	46	IM	Bone	Positive	CR	67
7	Male	43	IM	Bone	Negative	CR	60
8	Male	45	IM	Bone	Negative	PR	74
9	Male	23	RM	Bone, lung	Positive	CR	128
10	Male	36	RM	Bone, lung, pleura	Negative	CR	101
11	Male	43	RM	Lung	Negative	CR	97
12	Male	40	IM	Liver, lung	Negative	CR	135

AEs are shown in Table 4. During induction therapy, the most common AEs ≥ grade 3 were leucopenia (39.5%), acne-like rash (11.6%), febrile neutropenia (14%), and thrombocytopenia (9.3%). Frequent grade 3/4 toxicities exceeded 10% of patients during CCRT, including oral mucositis (39.1%), dermatitis (in-field) (26.1%), leukopenia (17.4%), acne-like rash (13%), and thrombocytopenia (13%). Severe (i.e., grade 3/4) toxicities during maintenance treatment were rare, including hand-foot skin reactions in one patient and hyperbilirubinemia in one patient, and these 2 patients discontinued treatment because of the toxic effects. No patients died during treatment or within 30 days of completion of CCRT. Except for some acne-like rash in patients with the novel regimen but not in patients with conventional regimens, the novel regimen did not result in increased AEs according to the toxicities grade classification.

**Table 4:** Adverse events during different periods of treatment in the study group.

	**Induction (*****N*** **=** **43)**		**CCRT (*****N*** **=** **34)**		**Maintenance (*****N*** **=** **15)**	
**Toxicity**	**Any grade**	**Grade ≥ 3**	**Any grade**	**Grade ≥ 3**	**Any grade**	**Grade ≥ 3**
Leukopenia	31 (72.1)	17 (39.5)	16 (47.1)	6 (17.6)	1 (6.0)	0
Acne-like rash	19 (44.2)	5 (11.6)	10 (29.4)	4 (11.8)	2 (13.3)	0
Dermatitis (in-field)	0	0	20 (58.8)	8 (23.5)	0	0
Nausea	18 (41.9)	0	6 (17.6)	3 (8.8)	2 (13.3)	0
Vomiting	6 (14.0)	0	3 (8.8)	2 (5.9)	1 (6.0)	0
Oral mucositis	8 (18.6)	0	22 (64.7)	13 (38.2)	2 (13.3)	0
Febrile neutropenia	6 (14.0)	6 (14.0)	4 (11.8)	3 (8.8)	0	0
Hyperbilirubinemia	3(7.0)	0	2(5.9)	0	2(13.3)	1(6.0)
Infusion reaction	3(7.0)	0	0	0	0	0
Infection	2 (4.7)	0	6 (17.6)	2(5.9)	0	0
Diarrhea	4 (9.3)	0	3 (8.8)	0	1 (6.0)	0
Premature heartbeat	1 (2.3)	0	1 (2.9)	0	0	0
Alopecia	10 (23.3)	0	5 (14.7)	1 (2.9)	0	0
Thrombocytopenia	5 (11.6)	4 (9.3)	6 (17.6)	4 (11.8)	0	0
Transaminitis	2 (4.7)	0	4 (11.8)	0	1 (6.0)	0
Anemia	3(7.0)	1 (2.3)	6 (17.6)	3 (8.8)	0	0
Hypokalemia	2 (4.7)	0	2 (5.9)	1 (2.9)	1 (6.0)	0
Peripheral neuropathy	0	0	17 (50.0)	1 (2.9)	1(6.0)	0
Hand-foot skin reaction	0	0	0	0	4 (26.7)	1 (6.0)
Dysphagia	0	0	12 (35.3%)	7 (20.6)		

**Figure 3:**
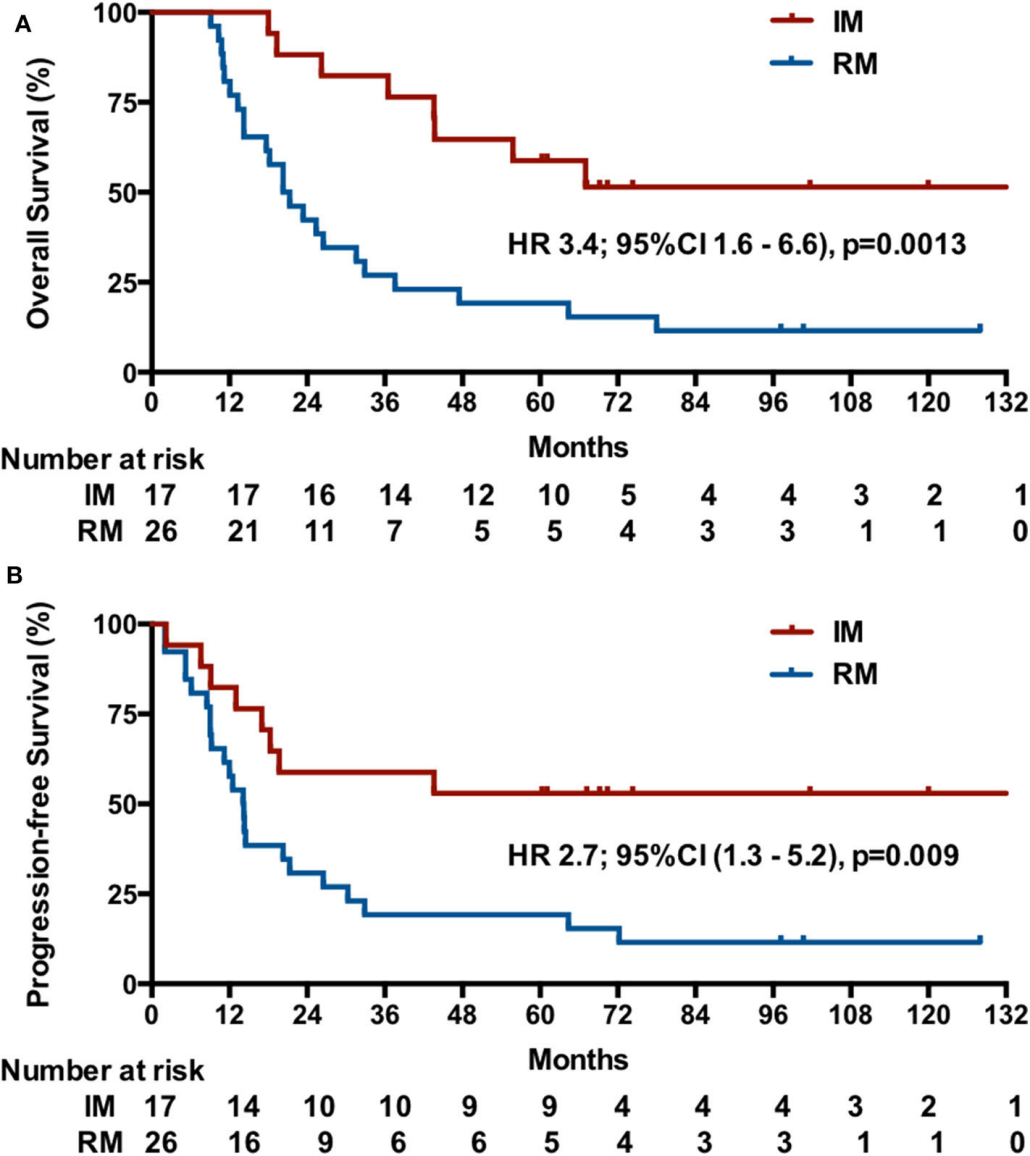
Kaplan-Meier estimates of OS **(A)** and PFS **(B)** among patients initially diagnosed with mNPC (IM) or NPC patients with first-relapse metastases after radiotherapy (RM).

The median OS was unreached (95% CI undefined; eight events) in patients with IM and was 20.3 months (95% CI, 13.8–26.8; 23 events) in patients with RM (HR, 3.4; 95% CI, 1.6–6.6, *p* = 0.0013; Figure 3A). In patients with IM, the median PFS was more than 44 months (eight events; [51.5% of deaths were in 44 mo]) vs. 12.5 months (95% CI, 11.2–17.0; 23 events) in patients with RM (HR, 2.7, 95% CI, 1.3–5.2; *p* = 0.009; Figure 3B).

**Figure 4:**
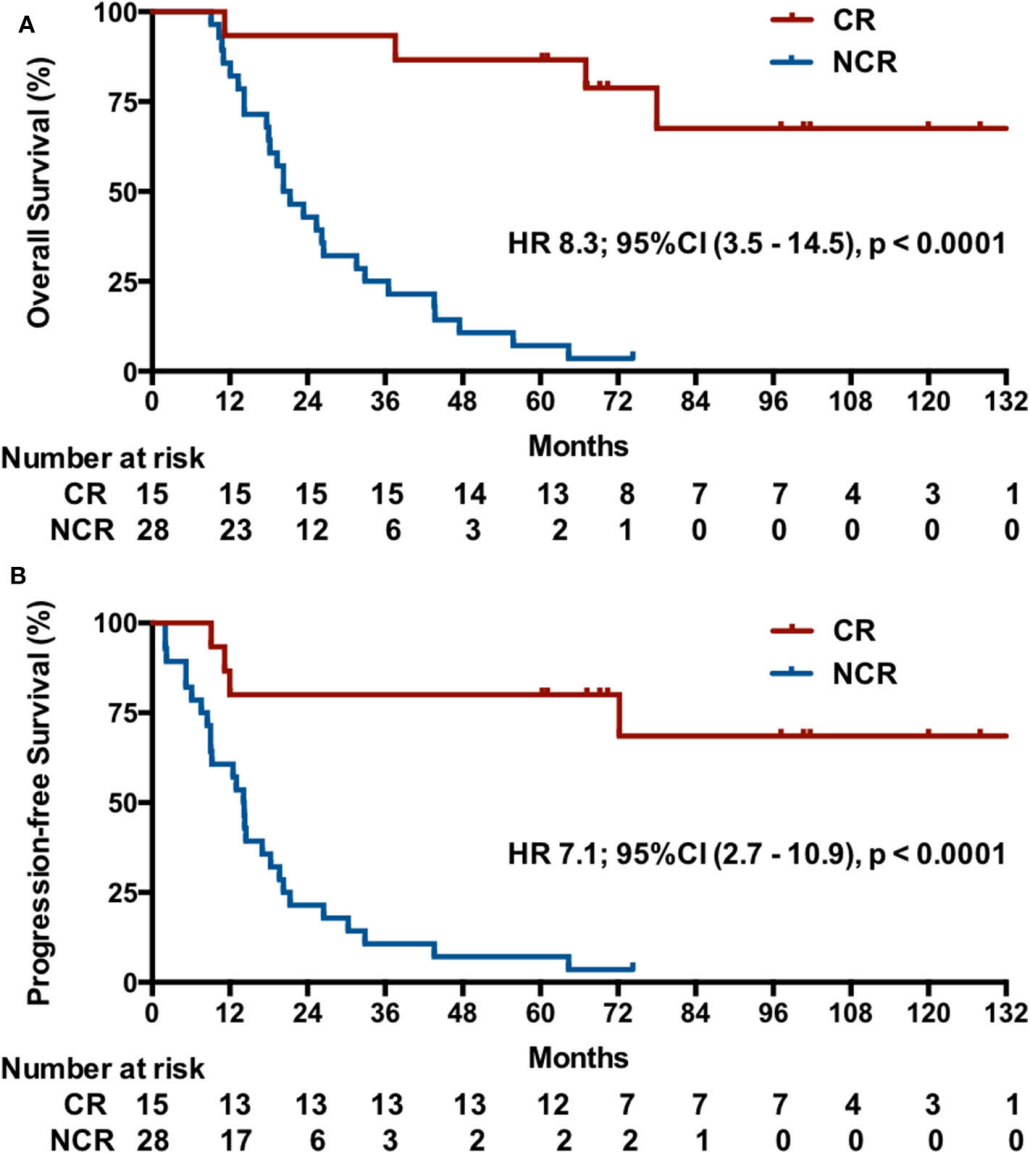
Kaplan-Meier estimates of the OS **(A)** and PFS **(B)** by CR after induction chemotherapy.

*Post-hoc* analysis showed that the IM group had a higher CR rate (9/17, 52.9%; 95% CI, 29.2–76.7%) compared with 23.1% (6/26; 95% CI, 6.9–39.3%) in RM patients (*p* = 0.045). Indeed, the 15 patients with a CR had a significant longer OS than these patients without a CR after induction chemotherapy (median OS, undefined vs. 20.3 months [95% CI, 15–25.6], *p* < 0.001), with a better OS at 2 years (93.3 vs. 42.9%) and 5 years (82.2 vs. 7.1%) and a lower risk of death (HR, 8.3, 95% CI, 3.5–14.5, *p* < 0.000; Figure 4A). Correspondingly, these patients also exhibited a better PFS (median PFS, undefined vs. 14.1 months [95% CI 11.8–16.4], HR 7.1, 95% CI, 2.7–10.9, *p* < 0.0001) and a higher 2-year PFS (80 vs. 21.4%) and 5-year PFS (80 vs. 7.1%) (Figure 4B).

## Discussion

Despite advances in radiotherapy and effective systemic agents during the past decade, the long-term survival of patients with mNPC remains poor. The standard first-line treatment of platinum-containing doublet regimens for mNPC is essentially palliative therapy. This new therapeutic strategy in our study yielded significantly long durations of OS and PFS (5-year OS, 33.2%; 5-year PFS, 29%). Moreover, further subgroup analyses suggested that patients who were not pretreated with radiotherapy achieved better outcomes than radiotherapy-pretreated patients. The 5-year OS and PFS were 54.4 and 51.5% in initially diagnosed mNPC patients, respectively. This finding may be associated with the history of radiotherapy. Previous ionizing radiation may increase chemotherapy resistance, as confirmed in prostate cancer and chronic myeloid leukemia (19, 20). A low survival rate in the contemporaneous controls was observed in our center (5-year OS, 10.9%; 5-year PFS, 0%), which was in accordance with previous reports. The favorable outcome of the novel regimen indicates the possible opportunity to completely cure chemotherapy-naïve mNPC, especially in patients with IM.

A long survival time is particularly prominent for patients who achieve a CR or PR of metastatic lesions after systemic chemotherapy (21). One study analyzed these different treatment combinations (induction, concurrent, and maintenance chemotherapy) and found that only induction-based chemotherapy was associated with significantly improved survival (22). In our study, the OR and CR rates after induction chemotherapy were 79.1 and 34.9%, respectively. Furthermore, 94% of patients with IMs achieved objective remission, and more than half of them exhibited CR after induction chemotherapy. Induction therapy consisting of cetuximab plus cisplatin and docetaxel in the regimen conferred a significant improvement in the response rate, especially the CR rate, vs. historic controls (OR rate, 60–74%; CR rate, 3–7%) (9) and contemporaneous controls (OR rate, 47%; CR rate, 3%) in our center. These results imply that adding cetuximab to induction chemotherapy improved chemotherapy outcomes. In fact, anti-EGFR monoclonal antibody therapy can improve the effect of chemotherapy or reverse resistance to the chemotherapy agent. Cetuximab was shown in a previous study to circumvent irinotecan resistance in irinotecan-refractory colorectal cancer (23). In metastatic/recurrent head and neck squamous-cell carcinoma (HNSCC) or squamous-cell lung cancer, the addition of these molecular-targeted agents, such as cetuximab, nimotuzumab, panitumumab, necitumumab, to platinum-based chemotherapy also improves the response rate and survival (13, 24–27). Chan et al. found a dose-dependent additive enhanced antitumor activity when cetuximab was combined with cisplatin or taxanes in NPC cell lines (28) and then confirmed its clinical activity in combination with carboplatin in heavily pretreated patients with mNPC (14).

Several studies have shown that radiotherapy to the primary tumor site combined with active systematic therapy can improve the survival of patients with stage IVc NPC (29, 30). Anti-EGFR-targeted agents have been demonstrated to improve the effect of chemoradiotherapy or to reverse radiotherapy resistance (12, 31, 32). The multicenter ENCORE study (33) and a phase 2 study (31) in Hong Kong Prince of Wales Hospital both showed prolonged 2-year PFS beyond 85% compared with historic data in patients with locoregional advanced NPC who received cetuximab-added chemoradiotherapy. During our study, among 34 patients who attained an objective response after induction therapy and continued to receive CCRT, 33 achieved further remission, and one case exhibited PD. Another anti-EGFR humanized antibody, nimotuzumab, also provided survival benefit when used concurrently with chemoradiotherapy in HNSCC (34, 35). Nevertheless, the addition of panitumumab to CCRT did not confer any benefit in HNSCC (36). The role of these EGFR antagonists in mNPC needs to be assessed in the future. The investigations in the studies above have demonstrated the safety and tolerability of cetuximab in patients with locoregionally advanced or recurrent and/or metastatic NPC. However, our study is the first to explore the addition of cetuximab to two processes of one regimen, i.e., induction and chemoradiation. There were few grade 3 skin reactions and no treatment-related mortalities or discontinuations of therapy reported during the entire treatment period. Importantly, in the last years local therapy of oligometastatic disease shows improvement of overall survival in several types of cancer. In our study patients also underwent local therapy of metastatic disease whenever possible. Therefore, not only systemic therapy but also local therapy may improve the overall survival. However, it required a further study to confirm the function of local therapy for residual metastatic foci after induction therapy.

In the present study we selected capecitabine but not cetuximab as maintenance therapy based on the following reasons: first, at present, fluorouracil or capecitabine plus cisplatin is one of the widely used regimens in patients with recurrent or metastatic NPC. Moreover, single-agent capecitabine as a maintenance treatment has already shown a favorable safety profile in other metastatic cancers (37, 38). Second, based on our clinical trial initiated by investigator rather than a company-sponsored study, it is difficult for most patients to afford the high cost of cetuximab for a long maintenance therapy. Last, capecitabine is more convenient for oral administration, which does not require weekly intravenous injection like cetuximab. However, our data showed that one-third of patients had PD during the oral administration of capecitabine as maintenance treatment, suggesting the need for further exploration of the role of this strategy. In addition, anti-PD-1 antibodies (39, 40) have shown promising antitumor activity (OR rate>20%) for multiply pretreated mNPC, which may be considered as another choice for maintenance therapy.

Our regimen was derived from this above evidence and showed good outcomes. Metastatic NPC appears to be incurable from the current literature. Few studies have reported the 5-year OS for mNPC, while patients with mNPC at initial diagnosis obtained a 54.4% 5-year OS rate in our study. Although few long-term survivors after various aggressive treatments were presented in a retrospective study (4), currently, no prospective study has reported a definite regimen that could result in a considerable long-term survival rate for mNPC. In our study, 15 patients (34.9%) who achieved long-term survival (>60 months), among whom, 12 were still alive with no evidence of disease at the 60 to 135-month follow-ups. Our data suggest a potential curative role for chemotherapy-naïve mNPC when the novel regimen is applied. To the best of our knowledge, this study is the first report of a series of long-term survivors with mNPC. Although this study was a non-randomized and single-armed phase II study trial, we have to realize that the novel study regime at the time of 2006 is a very bold, new and high-intensity scheme with the attempt to achieve an expected long survival. Considering this limitation, we have currently initiated a randomized multi-center phase 3 trial (NCT02633176) in 2015 to further investigate this topic.

## Data Availability Statement

The datasets generated for this study are available on request to the corresponding author.

## Ethics Statement

The studies involving human participants were reviewed and approved by the ethics committee of Sun Yat-sen University Cancer Center. The patients/participants provided their written informed consent to participate in this study.

## Author Contributions

TLi: conception and design. HHu, YH, SL, and TLi: development of methodology. MZ, HHu, XL, YH, CC, XFa, SL, and HHo: acquisition of data. MZ, HHu, XL, ZW, CG, SL, XFu, TLu, and TLi: analysis and interpretation of data. MZ, TLi, HHu, and XL: writing, review and/or revision of the manuscript. HHu, YT, and TLu: administrative, technical, or material support. TLi and TLu: study supervision. All authors contributed to the article and approved the submitted version.

## Conflict of Interest

The authors declare that the research was conducted in the absence of any commercial or financial relationships that could be construed as a potential conflict of interest.

## Supplementary Material

Supplement Figure 1 Kaplan-Meier estimates of the OS **(A)** and PFS **(B)** among patients receving the novel regimen or conventional treatment.

Supplement Table 1 Demographic and clinical characteristics of the patients receiving novel regimen and conventional regimen.
