# Supplementary figures and images for: Long-Term Survival of Patients With Chemotherapy-Naïve Metastatic Nasopharyngeal Carcinoma Receiving Cetuximab Plus Docetaxel and Cisplatin Regimen
Source: Front Oncol. 2020 Jun 19;10:1011. doi: 10.3389/fonc.2020.01011 (PMC7319102; doi:10.3389/fonc.2020.01011)

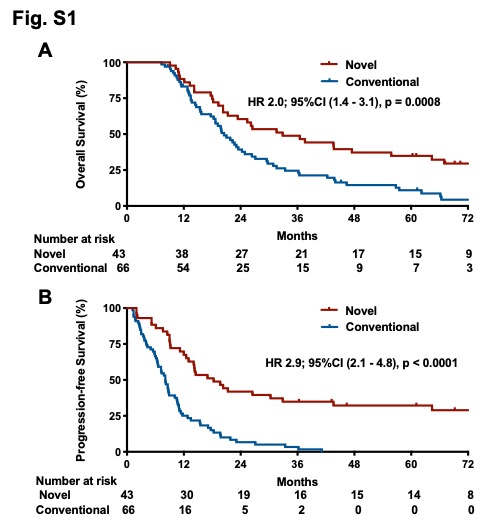

Supplement: Supplement Figure 1 — Kaplan-Meier estimates of the OS (A) and PFS (B) among patients receving the novel regimen or conventional treatment. [file Image_1.JPEG]
