# Supplementary material for: Long-Term Survival of Patients With Chemotherapy-Naïve Metastatic Nasopharyngeal Carcinoma Receiving Cetuximab Plus Docetaxel and Cisplatin Regimen
Source: Front Oncol. 2020 Jun 19;10:1011. doi: 10.3389/fonc.2020.01011 (PMC7319102; doi:10.3389/fonc.2020.01011)
Supplement: Supplement Table 1 — Demographic and clinical characteristics of the patients receiving novel regimen and conventional regimen. [file Table_1.DOCX]

| Supplement table 1: Demographic and clinical characteristics of the patients receiving novel regimen and conventional regimen | | | |
| --- | --- | --- | --- |
| Characteristics | Novel group(n=43) | Conventional group (n=66) | p-value |
| Gender |  |  | 0.874 |
| Female | 7 (16.3) | 10 (15.2) |  |
| Male | 36 (83.7) | 56 (84.8) |  |
| Age, years* |  |  | 0.334 |
| Median | 43 | 45.2 |  |
| Range | 23-63 | 19-76 |  |
| ECOG performance status |  |  | 0.675 |
| 0 | 14 (32.6) | 19 (28.8) |  |
| 1 | 29 (67.4) | 47 (71.2) |  |
| Histology |  |  | 0.97 |
| WHO type 2 | 4 (9.3) | 6 (9.0) |  |
| WHO type 3 | 39 (90.7) | 60 (91.0) |  |
| EBV-DNA status |  |  | 0.653 |
| Positive* | 32 (74.4) | 50 (75.8) |  |
| Negative | 11 (25.6) | 16 (24.2) |  |
| Number of metastatic organs |  |  | 0.524 |
| 1 | 27 (62.8) | 39 (59.1) |  |
| 2 | 8 (18.6) | 18 (27.3) |  |
| ≥3 | 8 (18.6) | 9 (13.6) |  |
| Sites of disease at registration |  |  | 0.331 |
| Distant lymph node | 7 (16.3) | 11 (16.7) |  |
| Bone | 32 (74.4) | 41 (62.1) |  |
| Liver | 14 (32.6) | 23 (34.8) |  |
| Lung | 11 (25.6) | 27 (40.9) |  |
| Others | 6 (14.0) | 5 (7.6) |  |
| Prior radiotherapy |  |  | 0.765 |
| Yes | 26 (60.5) | 38 (57.6) |  |
| No | 17 (39.5) | 28 (42.4) |  |
| Data are presented as a number (percentage) unless otherwise indicated. *Positive: EBV- DNA copies ≥10^3^ copies/mL. | | | |
